# Spatial insights into immunotherapy response in non-small cell lung cancer (NSCLC) by multiplexed tissue imaging

**DOI:** 10.1186/s12967-024-05035-8

**Published:** 2024-03-04

**Authors:** James Monkman, Afshin Moradi, Joseph Yunis, Geoff Ivison, Aaron Mayer, Rahul Ladwa, Ken O’Byrne, Arutha Kulasinghe

**Affiliations:** 1 Faculty of Medicine, Frazer Institute, The University of Queensland, 37 Kent Street, Woolloongabba, Brisbane, QLD 4102 Australia; 2 Faculty of Medicine, Ian Frazer Centre for Children’s Immunotherapy Research, Children’s Health Research Centre, The University of Queensland, Brisbane, QLD Australia; 3 Enable Medicine, Menlo Park, CA USA; 4 Princess Alexandra Hospital, Brisbane, QLD Australia

## Abstract

The spatial localisation of immune cells within tumours are key to understand the intercellular communications that can dictate clinical outcomes. Here, we demonstrate an analysis pipeline for highly multiplexed CODEX data to phenotype and profile spatial features and interactions in NSCLC patients that subsequently received PD1 axis immunotherapy. We found that regulatory T cells (Tregs) are enriched in non-responding patients and this was consistent with their localization within stromal and peripheral tumour-margins. Proximity-based interactions between Tregs and both monocytes (p = 0.009) and CD8^+^ T cells (p = 0.009) were more frequently found in non-responding patients, while macrophages were more frequently located in proximity to HLADR^+^ tumour cells (p = 0.01) within responding patients. Cellular neighbourhoods analysis indicated that both macrophages (p = 0.003) and effector CD4^+^ T cells (p = 0.01) in mixed tumour neighbourhoods, as well as CD8^+^ T cells (p = 0.03) in HLADR^+^ tumour neighbourhoods were associated with favorable clinical response. Evaluation of the inferred regulatory functions between immune cells relative to the tumour suggested that macrophages exhibit an immunosuppressive phenotype against both CD4^+^ and CD8^+^ T cells, and that this association scores more highly in ICI refractory patients. These spatial patterns are associated with overall survival in addition to ICI response and may thus indicate features for the functional understanding of the tumour microenvironment.

**Supplementary Information:**

The online version contains supplementary material available at 10.1186/s12967-024-05035-8.

## Introduction

Non-small cell lung cancers (NSCLC) account for 85% of lung cancers, which have the highest morbidity and mortality rates of all cancer globally. Whilst platinum-based therapies have been standard of care, the past decade has seen the increasing clinical adoption of immune checkpoint inhibitors (ICIs) that enhance the adaptive immune system's cytotoxic T cell response to target tumour cells. These therapies have led to durable benefits in a small subset of NSCLC patients [1–3], however, the reason for this phenomenon is poorly understood. This has spurred a field of investigation into the immune microenvironment of lung tumours to determine the biomarkers and cellular phenotypes that are predictive of response to ICI therapy [4–6].

PD-L1 expression and tumour mutation burden (TMB) as a measure of neoantigen load form current companion diagnostic assays for ICI treatment, however these assays suffer from relatively poor performance in stratifying responsive patients [7, 8]. Indeed, meta-analysis of the field across 10 tumour types in 8135 patients has shown that PD-L1 IHC (AUC 0.65) and TMB (AUC 0.69) fall behind multiplex immunofluorescence methods (AUC 0.79) in their ability to accurately predict patient ICI response [9]. Moreover, this suggests largely that the efficacy of the ICIs is not tied directly to PD-L1 directed immune evasion nor tumour antigenicity but rather other phenotypic properties of tumour tissues that are being systematically discovered through multiplexed imaging techniques [10, 11].

Spatial metrics describing the geographical associations between cells are providing increasing depth to our ability to quantify and compare tissue composition, and are poised to deeply aid our understanding of the cellular and functional architecture of tumour tissue. Models from multispectral imaging data that integrated cellular density and nearest neighbour distance have implicated the role for several effector-lymphocytic populations in the survival of patients treated by curative resection [12, 13]. Immune scores based on quantitative analysis of CD8 and PD-L1 [14], and CD8 and CD163^+^ macrophages [15] have shown utility in predicting immunotherapy outcome in multiple cancer types. Additionally, tumour infiltrating lymphocytes (TILs) identified from H&E have been clustered by the morphological properties of their spatial neighbour TILs, to computationally phenotype each TIL into one of 8 niche clusters. Signatures of these clusters were associated with outcome in NSCLC subtypes under different treatment regimens [16]. Imaging mass cytometry (IMC) of immune subtypes in NSCLC has indicated cell distributions, interactions and neighbourhood enrichment in both histology and clinical covariates in a chemotherapy cohort [17]. This approach was extended to a parallel assay of 27 ICI patients and indicated that chemokine CXCL13 promotes memory CD8^+^ and CD4^+^ T cells, which are activated upon PD1 blockade. CXCL13 also acts to reduce immunosuppressive CCR2 + monocytes in tumours, promoting tumour clearance. [18].

Despite these very recent findings, application of high-plex based cell typing coupled with spatial metrics beyond cellular density have been limited in the field of ICI biomarker discovery. To this end, CO-Detection by indEXing (CODEX) staining was applied to NSCLC tissues from a retrospective ICI cohort to examine the immune cell composition and spatial features that associate with ICI response. The analysis of multiplex images generated by CODEX is complex. It requires coordination of both supervised and unsupervised analytical steps to accurately assign genuine cell phenotypes from single cell expression data of cohorts of FFPE samples with varying expression levels [19]. This approach differs significantly from trained thresholds that may be applied to optimized, amplified multispectral data [11, 12]. Indeed, no single tool offers a turn-key solution for image QC, cell segmentation, data integration and normalization, parameter optimization, cell phenotyping and spatial analysis to identify features associated with binary and time to event clinical outcomes [20]. Current methods to assign cell types are based largely on unsupervised clustering using proprietary algorithms [21–23], while more recent novel machine learning methods including Astir [24], CELESTA [24], and STELLAR [25] remain difficult to implement due to their sensitivity to staining intensity, stringent a-priori rules or requirement for accurate training data.

Here we developed a bespoke analytical pipeline that employed open source and published packages to derive insights from the CODEX technology in a novel ICI therapy treated cohort. Following cell phenotyping and cell proportion analysis in spatial compartments, we employed publicly available metrics including an interaction metric in the Scimap package, cellular neighbourhood detection and cell type enrichment within these communities [23], as well as further spatial metrics that putatively identify functional implications from 3-way proximity ratios [26].

## Materials and methods

### Patient information and TMA construction

Pre-treatment tissue microarrays (TMAs) were constructed from resected NSCLC tissues in collaboration with TriStar Technology Group (USA). Tissues were obtained from patients who were subsequently treated with immune checkpoint immunotherapy (ICI) in advanced or metastatic setting following recurrence after surgery. Tissues were collected between 2009 and 2018, with censor times ranging between 258 and 3243 days. TMA consisted of 42 patient cores, 6 of which were excluded due to poor tissue quality, and response information was not available for one patient (Table 1). TriStar pathologists reviewed whole sections prior to coring representative tumour regions that avoided benign tissue, and included stroma and tumour around invasive margins. TMAs consisted of single 1 mm cores per tumour sample. All patient clinicopathological, treatment, ICI response and survival parameters were provided by TriStar's medical teams. Clinical endpoints included ICI response according to RECIST 1.1 criteria and overall survival. Thirty-nine percent of patients were classified as responsive (R), while sixty-one percent were non-responsive (NR). Anti-PD-1 therapies Nivolumab and Pembrolizumab comprised 93% of treatments, with one patient receiving anti-PD-L1 agent Durvalumab. 93% and 38% patients remained alive at follow up time, for responsive, and non-responsive groups, respectively. The cohort contained both squamous and adenocarcinoma NSCLC histology. This study has Queensland University of Technology (QUT) Human Research Ethics Committee approval (UHREC #2000000494) and University of Queensland ratification.

**Table 1:** Patient characteristics

	Responder, n = 14	Non-responder, n = 21
Sex		
F	5 (36%)	9 (43%)
M	9 (64%)	12 (57%)
Stage at diagnosis		
I	4 (29%)	8 (38%)
II	3 (21%)	3 (14%)
III	5 (36%)	7 (33%)
IV	2 (14%)	3 (14%)
Histology		
Adenocarcinoma	12 (86%)	12 (57%)
Squamous Cell Carcinoma	2 (14%)	9 (43%)
IO treatment		
DURVALUMAB	1 (7.1%)	0 (0%)
NIVOLUMAB	10 (71%)	18 (86%)
PEMBROLIZUMAB	3 (21%)	3 (14%)
Status		
Alive	13 (93%)	8 (38%)
Deceased	1 (7.1%)	13 (62%)

### CO-detection by indEXing (CODEX) staining

CODEX (Akoya Biosciences, US) staining was performed by Enable Medicine, US, as previously described [27]. FFPE TMA sections were mounted on 20 mm x 20 mm poly-lysine treated coverslips. Coverslips were rehydrated and subjected to heat induced epitope retrieval. Coverslips were incubated with 190uL of antibody cocktail solution containing 36 antibodies for 3 h at room temperature in a humidity chamber. This was followed by several cycles of washing and fixation steps. Antibody barcode, dilution, and imaging cycle were performed as shown in supplementary (Supplementary Table 1). Antibody optimisation and validation for CODEX was performed by Enable Medicine. For a more detailed protocol, see reference ^[27]^. Coverslips were imaged on an inverted fluorescence microscope (Keyence BX-810) using a Plan Apo 20 × 0.75 NA objective (Nikon). The Codex imaging cycles were performed using a Codex Instrument (Akoya Biosciences). Large regions were broken up into tiled subregions, and five z-stack slices with a step size of 1.5 µm were acquired.

Images were deconvolved and pre-processed using image CODEX pre-processing pipeline (Enable Medicine, US). Briefly, background signal was removed from the image by using a computationally aligned blank acquisition cycle as a reference channel. Then, image deconvolution was performed for each biomarker image z-stack, and the best focus was chosen using an extended depth of field algorithm. Finally, the individual tiles were aligned and stitched together, and all channels were stacked. OME-TIFF were output for image analysis.

### Image analysis

Images were imported into Qupath [28] and segmented with Cellpose [29] plugin on DAPI2 channel using 'cyto2' pretrained model with ‘.cellExpansion’ = 3 µm and ‘.cellConstrainScale’ = 1.5. The performance of cell segmentation was visually inspected. Poor quality TMA cores that were fragmented, folded or necrotic with regions of high non-specific fluorescence were excluded. An artificial neural network (ANN) pixel classifier was trained on pan-cytokeratin signal to define a tumour/stroma annotation mask which captured pan-cytokeratin positive pixels as ‘tumour’ regions. A minimum 100µm2 threshold was applied such that only tumour nests larger than this were annotated as ‘tumour’. The tumour annotation was expanded by 30 µm to define a peripheral tumour 'margin'. Cell metrics including universally unique identifier (UUID) codes, spatial coordinates, nuclear size, and median cell expression for each channel was output for analysis in python. Cell classifications generated by subsequent unsupervised clustering were imported back into Qupath and matched by their UUID for visual inspection and ground truth QC. Cell visualisations were generated in Qupath.

### Cell classification

Expression matrices and cell metadata were imported into Anndata [30, 31] format for QC/preprocessing, clustering and cell phenotyping. Artifactual nuclei were removed by applying a minimum median DAPI signal threshold, followed by size exclusion < 10µm2 and > 220µm2. Nuclei smaller than 10um tended to be fragmented, while cells larger than 220um tended to be aggregated or dividing nuclei. Raw data contained thirty-six markers. Markers that possessed low signal to noise or high nonspecific background were excluded. Expression matrices were arcsinh (cofactor 150) transformed, scaled within columns (markers), then scaled across rows (cells) according to recommended methods for CODEX pre-processing [19]. Data was then integrated using the Scanpy integration of Harmonypy [32] by TMA core and adjusted PCs used to cluster data with Phenograph [33] using k = 30 and leiden r = 2. Leiden resolutions 1–4 were assessed and resolution two was chosen empirically to resolve the most meaningful cell types. Clustering included twenty-five markers, PanCK, CD117, Ki67, CD45, CD20, CD3e, CD4, CD45RO, CD45RA, CD8, CD107a, CD44, FoxP3, CD25, CD197, CD11b, CD14, CD15, CD68, HLA-DR, CD141, CD31, CD34, Podoplanin and nuclear area (µm^2^) to aid discrimination of tumour cells from stromal cells. Cell typing was performed using canonical cell-type markers, and Phenograph resolved functional subsets in single iteration of clustering. Cell typing resolved B cells (CD45^+^, CD45RA^+^, CD20^+^), Blood vessels (CD31^+^, CD34^+^), Lymphatics (Podoplanin^+^), CD4 cells (CD45^+^, CD3^+^, CD4^+^, CD45RO^+^), CD8 cells (CD45^+^, CD3^+^, CD8^+^, CD45RO^+^), granulocytes (CD15^+^, CD141^+^, CD11b^+^), lymphocytes (CD45^+^), macrophages (CD68^+^, CD107^+^, CD14^+^), mast cells (CD117^+^), Monocytes (CD14^+^), proliferating lymphocytes (CD45^+^, Ki67^+^), stroma (Vim^+^), Treg (CD45^+^, CD4^+^, FoxP3^+^, CD25^+^), effector CD4 cells (CD45^+^, CD4^+^, HLADR^+^, CD197^+^), and CCR7^+^ CD8 cells (CD45^+^, CD45RO^+^, CD8^+^, CD197^+^). PanCK^+^ cells clustered into HLADR^+^, CD44^+^ or Ki67^+^ subsets. Cell type clusters were merged for further analysis.

### Cell analysis

Cell frequency was defined by summing the instances of each cell class and normalizing to the total cell count for each core or tumour/stroma/tumour margin region to obtain cell percentages. Interaction analysis was performed using the spatial_pscore function in Scimap (https://github.com/labsyspharm/scimap) with method = radius, radius = 20 µm, and knn = 3 for all permutations of pairwise combinations. Proximity density metric in spatial_pscore function was defined by the number of pairwise interactions divided by the number of cells of that cell-pair per sample, effectively scoring each sample by the proportion of a given cell pair occurring in a 20 µm radius.

### Neighbourhood analysis

Neighbourhoods were defined using the neighbourhood identification pipeline (https://github.com/nolanlab/NeighborhoodCoordination) [23]. For each cell, the cell types of the 10 nearest neighbours were assigned as the features of that cell. These features were then run through an unsupervised KNN algorithm and assigned to 10 clusters. The choice of 10 nearest neighbours and 10 clusters was chosen heuristically according to published datasets [23, 34]. Thus each cell was assigned to a cellular ‘neighbourhood’ that was a product of the cells most frequently found in its proximity. Differential cell type enrichment within each neighbourhood was performed using the linear model in the cell type differential enrichment pipeline[23], which scores the fold change and significance of cell types within neighbourhoods by a binary variable.

### SpatialScore analysis

SpatialScore was assessed as previously published (https://github.com/nolanlab/SpatialScore) [26] using method 1 to generate mean spatial ratios per sample between permutations of sets of 3 cells.

### Statistics

T-tests were performed between response status groups for each of these metrics (Additional file 3: Table S2). P values shown throughout are not adjusted for multiple testing, and were not significant following adjustment. Metrics were also assessed for OS associations by Cox proportional hazards models as continuous variables, as well as median cut point Kaplan Meier estimates (Additional file 4: Table S3).

## Results

### Multiplex image analysis workflow

**Fig. 1:**
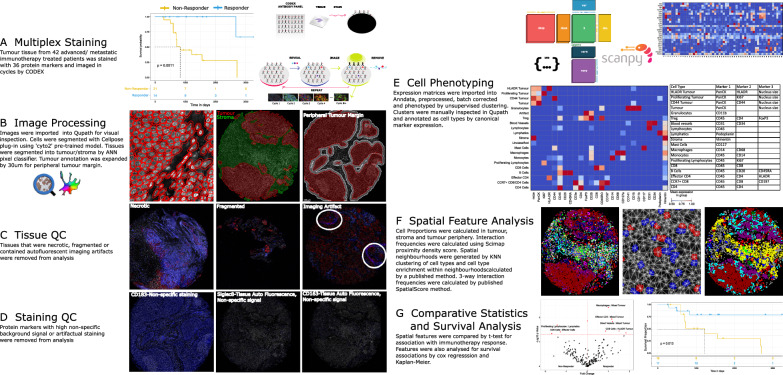
Analysis workflow for CODEX imaging data. **A** CODEX cyclical staining was performed on an initial cohort of 42 ICI treated patients. Significant differences in overall survival were evident between patient response groups **B** Images were imported into Qupath and segmented with Cellpose. A pixel classifier was trained on cytokeratin to create a tumour mask. This mask was expanded to capture cells at the tumour periphery. **C** Images were visually inspected for QC, and six cores that were fragmented, necrotic or contained imaging artifacts were excluded. Response information was not available for one patient. **D** Marker QC was performed and markers with non specific signal, or artifactual staining were excluded. **E** Cell phenotyping was performed in python by unsupervised clustering and cell annotation by canonical marker expression. **F** Spatial metrics were generated, including cell densities, pairwise cell–cell interaction, neighbourhood detection and cell type enrichment within these neighbourhoods, as well as published SpatialScore metric. **G** These spatial metrics were analysed for differences between ICI response groups and patient survival times

The analysis pipeline was developed and implemented as shown in Fig. 1. The ICI NSCLC cohort (Fig. 1A) was stained by CODEX. Cell segmentation was performed and cells were classified spatially within tumour, stroma and the peripheral tumour margin regions (Fig. 1B). Images were visually inspected to ensure tissue was of sufficient quality for analysis (Fig. 1C), leading to the exclusion of six cores that were either fragmented, out of focus portions or necrotic (Additional file 1: Fig. S1A). Response information was not available for one patient, leading to a final cohort of 35 patients. Staining quality was visually assessed (Fig. 1D) and markers with non-specific staining or low signal that corresponded with tissue autofluorescence were excluded, resulting in a final panel of 25 markers (Additional file 1: Fig. S1B, C). Cell phenotyping was performed by unsupervised clustering according to canonical marker expression (Fig. 1E). These cell phenotypes were then analysed by subsequent spatial techniques (Fig. 1F) for comparison against clinical variables (Fig. 1G.)

### Cell phenotyping and cell proportion analysis

**Fig. 2:**
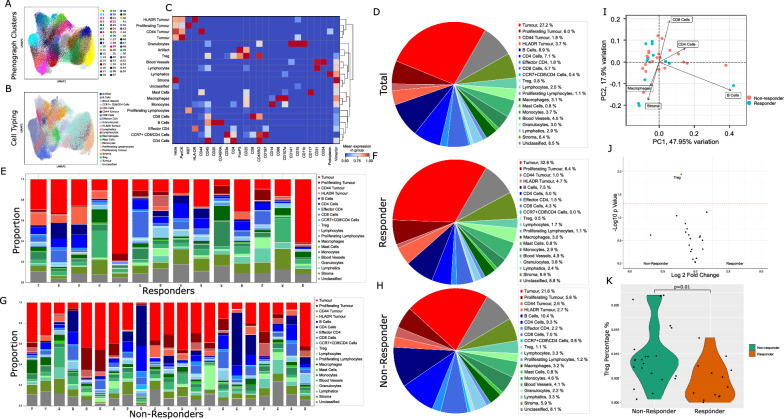
Cell phenotyping and total cell proportion analysis. **A** 55 clusters were resolved by Phenograph at leiden resolution 2 following integration by Harmonypy. **B** Annotated clusters were merged into 19 cell types. **C** Cell types were defined by canonical marker expression. **D** Over 90% of 210,945 cells in total cohort were phenotyped. **E**, **G** TMA cores consisted of heterogenous proportions of each cell type. **F**, **H** Analysis of cell types by outcome did not show clear differences between patient groups. **I** Principal component analysis indicated that sample variance was driven by proportions of CD4^+^, CD8^+^, macrophage and B immune cells. J,K) Differential enrichment indicated higher levels of Treg cells in non-responding patients. Significant results p < 0.05 in volcano shown in red. Not adjusted for multiple testing

Unsupervised clustering of 210,945 cells from 35 TMA cores resulted in 55 clusters that were phenotyped (Fig. 2A) according to their predominant canonical marker expression (Additional file 1: Fig. S2a, b). Clusters were annotated and merged into 19 cell types (Fig. 2B, C, Additional file 1: Fig. S3), including B cells, Blood vessels, Lymphatics, CD4^+^ T cells, CD8^+^ T cells, granulocytes, lymphocytes that localized to CD31^+^ blood vessels, macrophages, mast cells, Monocytes, proliferating lymphocytes, stroma, Tregs. Phenotypic subsets included effector CD4^+^ T cells, and CCR7^+^ CD8^+^ T cells which colocalized with aggregates of CD4^+^ T cells. Tumour cells clustered into discrete subsets expressing either HLADR, CD44 or proliferation marker Ki67.

The cellular content of each core was first assessed to determine cell types which may associate with outcome to ICI therapy. Over 90% of the cellular composition of the cohort was phenotyped (Fig. 2D), allowing for dissection of the cell-type proportion of each core (Fig. 2E, G) for potential association with patient response to PD1 blockade (Fig. 2F, H). While cellular composition of each core did not readily discern therapy response, levels of key immune cells including CD8^+^ T cells, CD4^+^ T cells, B cells and macrophages were the major factors of PCA component loadings (Fig. 2I). Treg cells were significantly elevated in non-responding tumours (Log2 FC 1.1, p = 0.01) (Fig. 2J, K, Additional file 2: Table S1).

**Fig. 3:**
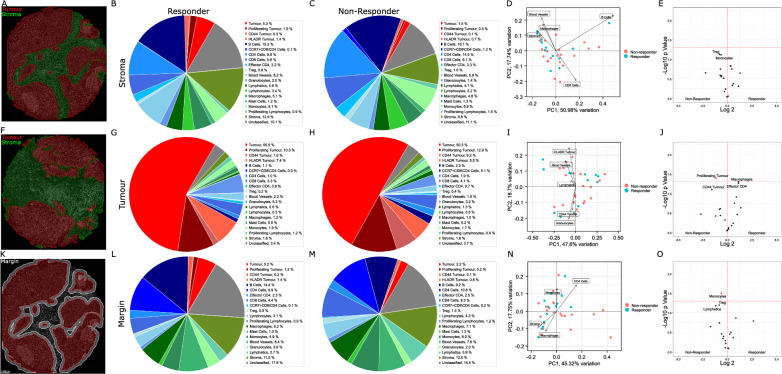
Cell proportion analysis within tissue compartments. **A**, **F** Representative TMA cores with tumour (red) and stroma (green) compartments. **B**, **C** Composition of the stromal compartment appeared similar in IO response groups. **D** Variance in patient stroma was largely driven by proportions of CD4^+^, B cells, blood vessels and macrophages. **E** Treg cells and monocytes were slightly elevated in non-responding patients. **G**, **H** Composition of the tumour compartment appeared similar in IO response groups. **I** Variance in tumour regions was driven by tumour cell CD44 and HLADR positivity, as well as levels of granulocyte infiltration. **J** Proliferating tumour cells were more abundant in non-responding patients. K) Tumour regions were expanded by 30um into the stroma to define a tumour margin (White). **L**, **M** Composition of the tumour margin appeared consistent between patient response groups. **N** Levels of CD4^+^, macrophages and monocyte cells were major components of variance in tumour interface. **O** Monocytes were enriched in tumour margin regions of non-responding patients. Significant results p < 0.05 in volcano shown in red. Not adjusted for multiple testing

The ability of immune cells to infiltrate into the malignant structure is requisite for anti-tumour activity. Each tissue was therefore further compartmentalized by applying a pan-cytokeratin mask to define tumour and stroma (Fig. 3A, F) regions. The tumour mask was expanded by 30 µm to define a tumour margin (Fig. 3K), representing the cellular content potentially acting directly at the tumour periphery. Compartmentalised cell proportion in stroma (Fig. 3 B, C), tumour (Fig. 3G, H) and tumour margin (Fig. 3L, M) indicated that similar heterogeneity in cell composition existed between patient response groups. Major stromal diversity was driven by levels of vascularization, CD4^+^ T cells and B cells (Fig. 3D), while variance in tumour content was attributed to the presence of HLADR or CD44 positive tumour cells (Fig. 3I). Notably, Tregs and monocytes were elevated in stroma (Fig. 3E) and margin regions (Fig. 3O) of non-responding patients, while proliferating and CD44 positive tumour cells appeared more abundant in tumour regions of non-responsive patients. A trend for increased macrophages and effector CD4^+^ T cell populations within tumour regions of patient responders also existed.

### Spatial cell–cell interactions

**Fig. 4:**
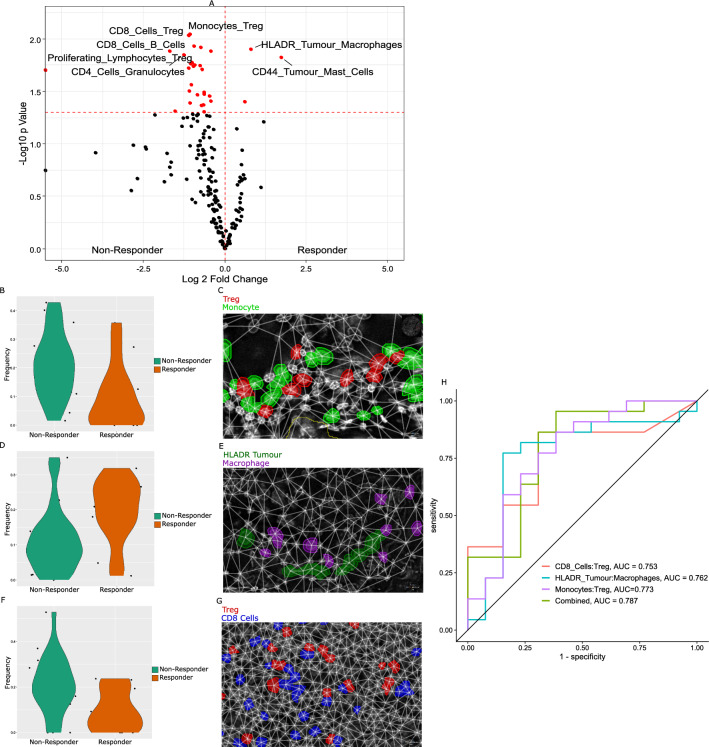
Cellular interaction analysis. **A** Volcano plot showing most significant cell–cell interactions enriched in patient response groups. Interactions were defined in Scimap by proximity of cell pairs within 20um normalised to the number of those cell types within each sample. These proximity densities were compared by T test between response groups, and unadjusted p values shown. **B** Violin plot of Treg: monocyte interaction frequency. p = 0.009. **C** Representative field of view of enriched Treg: monocyte interactions. **D** Violin plot of macrophage: HLADR^+^ tumour cell interaction frequency. p = 0.01 **E** Representative field of view of enriched macrophage: HLADR^+^ tumour cell interactions. **F** Violin plot of Treg: CD8^+^ cell interaction frequency. p = 0.009 **G** Representative field of view of enriched Treg: CD8^+^ interactions. **H** ROC curve indicating AUC of generalised linear model for binary classification of patient response by most significant interactions as well as when combined into multivariate model. Significant results p < 0.05 in volcano shown in red. Not adjusted for multiple testing

Tissue composition yields only partial insights into the cellular phenotypes responsible for anti-tumour immune responses. Spatial analyses were therefore applied to derive functional cell–cell relationships in tissue cores. The density of pairwise cell interactions were assessed using Scimap [35], where the frequency of interacting cells within a 20 µm radius per sample were normalized to the number of cells of that given pair contained in the sample (Fig. 4 A). This provides a per sample score for the frequency of each pairwise interaction, allowing ranking and comparison. Notably, this approach detected the enrichment of Treg proximity with both monocytes (Fig. 4B, C) (p = 0.01) and CD8^+^ T cells (Fig. 4F, G) (p = 0.01) in non-responder group, while macrophages appeared more frequently associated with HLADR^+^ tumour cells (Fig. 4D, E) (p = 0.012) in the patient responder group. Independently, in a logistic regression, these features had classification accuracies ranging from AUC 0.753 to AUC 0.773 (Fig. 4H), while collectively, a multivariate logistic regression with combined features yielded a model with AUC 0.787 classification accuracy (Fig. 4H), improved over independent features alone. Such models are however, likely overfitted due to the sample size of the cohort.

### Cellular neighbourhoods

**Fig. 5:**
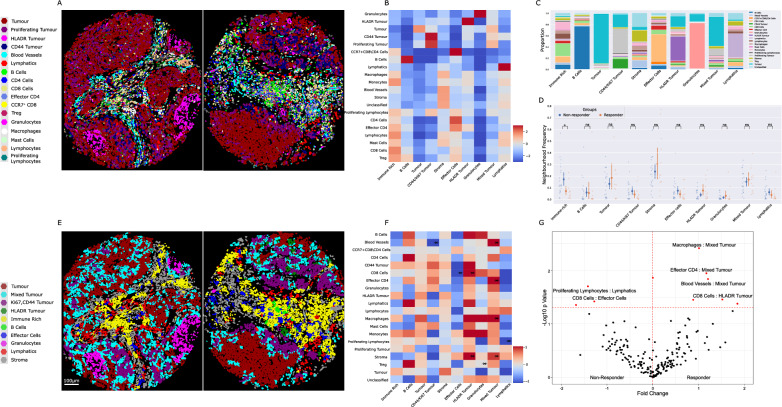
Cellular neighbourhoods and their cell type enrichment. **A** Representative images of cell types in tissue cores. **B** Cell types were clustered into unsupervised cellular neighbourhoods according to their nearest neighbours. Heatmap shows abundance of cell types in each neighbourhood **C** Proportion of cell types in each cellular neighbourhood. **D** Frequency of each neighbourhood in tissue cores was asessed and compared by T test between patient response groups. **E** Representive images of cellular neighbourhood composition in matched cores to A. **F** Differential enrichment of cell types in each neighbourhood by response groups were performed. Heatmap shows scaled change of cell types in each neighbourhood by patient response groups. **G** Volcano plot showing fold change of significant changes in cell type enrichment in each neighbourhood. Significant results p < 0.05 in volcano shown in red. Not adjusted for multiple testing

Functional properties of tissues are defined by both the activity of individual cells and the communities they form [23]. Unsupervised spatial clustering of cell types (representative Fig. 5A) by their 10 nearest neighbours was performed to yield cellular neighbourhoods (representative Fig. 4E) [23]. Such clustering yielded phenotypic communities (Fig. 5B), including immune cell rich, B cell, Tumour, CD44/Ki67 tumour, HLADR tumour, mixed tumour, effector cells, granulocytes, lymphatics, and stromal neighbourhoods, and were comprised of distinct proportions of each cell type (Fig. 5C). Assessment of tissues for the relative composition of each neighborhood indicated that immune rich neighbourhoods composed of monocytes, CD4^+^ T cells, CD8^+^ T cells, mast cells and Tregs were enriched in non-responsive patients (p = 0.018) (Fig. 5D).

Changes in the phenotypic composition of these neighbourhoods (Fig. 5F) may indicate functional dysregulation at the macroscopic level, including the propensity for immune activation upon ICI treatment. Data from this cohort suggested that the increase of macrophage (p = 0.003) and effector CD4^+^ T cells (p = 0.01) within mixed tumour (Ki67^+^/CD44^+^) neighbourhoods, as well as increased CD8^+^ T cells (p = 0.03) within HLADR^+^ tumour neighbourhoods were positively associated with ICI response (Fig. 5G).

### Spatial score–macrophages are immunosuppressive

**Fig. 6:**
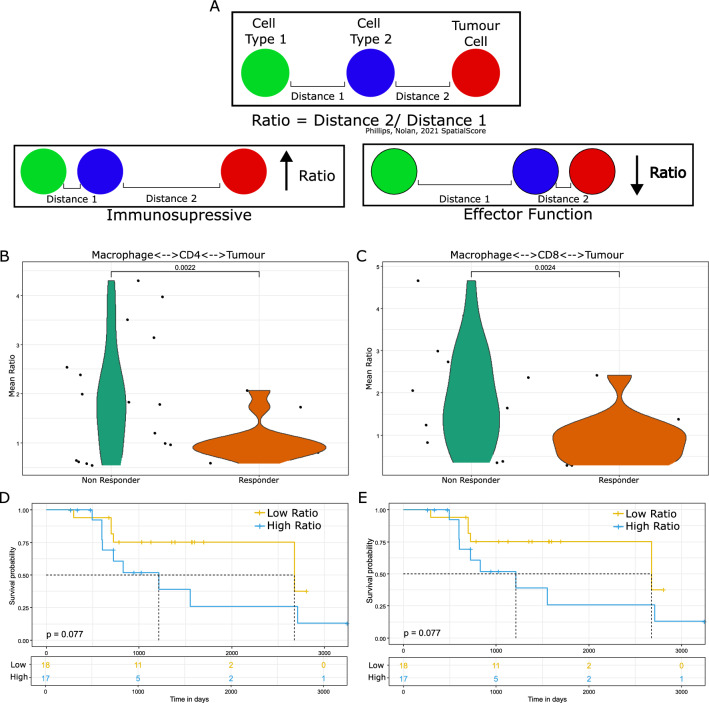
Spatial Score. **A** Schematic describing the published SpatialScore metric of implied immunosuppressive or effector activity by the ratio of immune cell proximity relative to tumour distance. **B** A higher ratio was observed for macrophages in proximity with CD4^+^ cells relative to tumour cells in non-responsive patients. p = 0.002 **C** A higher ratio was observed for macrophages in proximity with CD8^+^ cells relative to tumour cells in non-responsive patients. p = 0.002 **D** The higher ratio for macrophages:CD4^+^ cells corresponded with poorer patient survival. **E** The higher ratio for macrophages:CD8^+^ cells corresponded with poorer patient survival. Not adjusted for multiple testing

Indications that immune function may be inferred from the spatial topography of immune cells relative to tumour cells is a recent finding within the field of spatial biomarker discovery. The previously published SpatialScore [26] method was applied here to examine the distance between tumour and immune cells (Distance 2, Fig. 6A), relative to the proximity of that immune cell's nearest neighbour (Distance 1, Fig. 6A), allowing the quantification of putative immune suppression or effector function. Interestingly, a higher ratio was observed in non-responding patients for the association of macrophages with both CD4^+^ T cells (Fig. 6B) and CD8^+^ T cells (Fig. 6C) cells relative to tumour proximity, consistent with an M2 immunosuppressive phenotype. This phenotype was further reflected in patient overall survival, where higher immunosuppressive ratios with CD4^+^ T cells (Fig. 6D), and CD8^+^ T cells (Fig. 6E) exhibited a trend with poorer overall survival.

### Survival

**Fig. 7:**
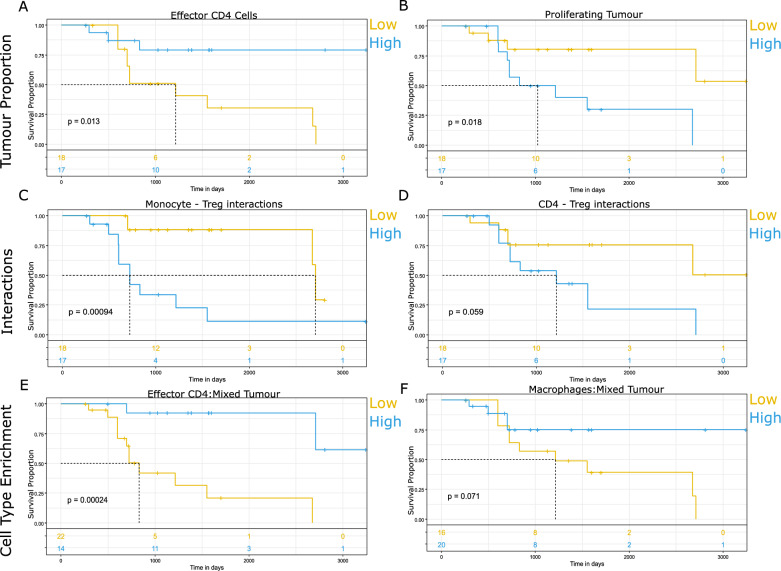
Kaplan Meier estimates of significant spatial features. **A** Higher levels of effector CD4^+^ T cells within tumour regions were associated with better survival. p = 0.013. B) Patients with increased proportions of proliferating tumour cells possessed poorer survival. p = 0.018. **C** Higher frequency of Tregs interacting with monocytes were associated with poorer survival. p = 0.001. **D** Higher frequency of Tregs interacting with CD4^+^ T cells were associated with poorer survival. p = 0.059. **E** Higher levels of effector CD4^+^ T cells within mixed tumour neighbourhoods were associated with better outcome. p = 0.0002. **F** Higher levels of effector CD4^+^ T cells within mixed tumour neighbourhoods were associated with better outcome. p = 0.071. Median thresholds were applied to categorise groups into high/low. Not adjusted for multiple testing

Features generated throughout the analysis were evaluated for associations with overall survival by cox proportional hazards (Additional file 3: Table S2) and at median thresholds by Kaplan–Meier estimates. Of note, higher effector CD4^+^ T cells within tumour regions were beneficial for patient survival (Fig. 7A), while proliferating tumour cells were associated with poorer survival (Fig. 7B). Higher frequency of Treg interactions with both monocytes (Fig. 7C) and CD4^+^ T cells (Fig. 7D) were also associated with poorer patient survival. In addition, mixed tumour neighbourhoods that were enriched in CD4^+^ effector T cells (Fig. 7E) or macrophages (Fig. 7F) exhibited better outcome.

## Limitations of study

The limitations of our study include a single-cohort TMA study with heterogenous tumour content that provide only a snapshot of the tumour tissue. Furthermore, limited clinicopathology information was available to discuss the potential impact of treatment history upon both immune composition and ICI outcome. The empirical testing of robust antibodies that successfully endure the disulphide reduction required for barcode conjugation, combined with a lack of fluorescent signal amplification mean that antibody validation is an ongoing aspect of CODEX based technology. Here, we employed a panel developed by a commercial CODEX supplier, Enable Medicine, based on the original publication [23], which had already undergone validation on positive and negative control tissues. External adoption and extension of such panels would again require validation of conjugation and epitope labelling, and may introduce batch effects between experimenters. As such, biases associated with current antibody availability and staining performance provide insights only into the particular cell types aimed to be assessed by the investigator, and our data here only indicates part of the immunome. Furthermore, aligning these results with studies that include more customized cell type definitions, namely by TSA multiplex fluorescence, is potentially confounding. While cell proportion is a common metric of cell density within tissue, our interaction and neighborhood metrics are only a subset of methods developed in spatial biology, and thus additional metrics may provide different results. The neighborhoods identified in this study are dependent upon the composition of this tumour cohort, and may be differently defined in other datasets. Thus, the data presented here provides a descriptive insight into a limited cohort, and is presented as an observational study of spatial metrics that require validation in additional immunotherapy cohorts.

## Discussion

Multiplexed single cell spatial technologies allow deep characterization of the cellular organization that occurs in tissues and are poised to reveal functional biology in the tissues that are taken routinely during biopsy or resection. Notwithstanding the limitations discussed above, we demonstrate here an approach to apply methods to generate spatial statistics for an important clinical question.

The data shown points to a prominent role for Tregs in ICI refractory disease. The immunosuppressive role of Tregs is well understood, however their relative abundance in non-responsive tumours coincides with the significant enrichment of their spatial proximity with both cytotoxic CD8^+^ T cells and monocytes. While immunoregulatory interactions between Tregs and CD8^+^ T cells are consistent with our understanding of T cell immunology, their potential role in influencing monocyte behavior may be a novel finding.

Monocytes are progenitor cells that migrate into damaged tissues and differentiate into dendritic or macrophage cell lineages. Tregs have been implicated to influence this maturation process by suppressing the expression of co-stimulatory and MHC-class II molecules and upregulating M2 macrophage marker CD206 [36, 37], thus directing monocyte maturation to an immunosuppressive polarised macrophage phenotype. This is consistent with further evidence in our data that the spatial association between macrophages and both CD4^+^ T cells and CD8^+^ T cells relative to their tumour proximity is also enriched in non-responsive patients. Taken together, these spatial relationships point to an axis of Treg induced macrophage M2 polarisation which may form a niche for ICI refractory disease.

Additionally, a discrete population of macrophages existed in our data that were aligned with anti-tumour activity within ICI responsive tumours. Macrophages possess dual roles as both phagocytic innate immune cells as well as antigen presenting cells for adaptive responses. Their interaction with antigen presenting HLADR^+^ tumour cells in responsive tumours is consistent with this role. Similarly, their enrichment in mixed tumour neighbourhoods in ICI responsive tumours suggests a tumour-killing role. While macrophage polarization was not directly observed through the expression of CD163, our spatial data implies dual roles for these key innate immune cells.

Activated effector CD4^+^ T cells expressing HLADR appear to also play a significant role in this cohort, with their enrichment beneficial in tumour regions as well as within in mixed tumour neighbourhoods. While ICIs specifically induce cytotoxic CD8^+^ T cell responses, the role for CD4^+^ T cell cooperation beyond traditional 'helper' activity and even CD4^+^ T direct cytotoxic activity is emerging [38]. Additionally, peripheral effector CD4^+^ T cells have been associated with PD1 efficacy [39]. Coexpression of CD197, the homing chemokine receptor CCR7, which directs T cells towards secondary lymph nodes, suggests that these effector CD4^+^ T cells are being encouraged to migrate out of the peripheral tumour tissue [40]. Taken together, this unique cell population provides an insight into novel roles for CD4^+^ T cells in anti-tumour activity.

## Conclusion

The richness of multiplex imaging data is positioned to vastly increase our understanding of tissue architecture as well as provide valuable information relevant to clinical decisions. While these findings require validation within additional cohorts and modalities, our data has allowed the novel observation of a number of phenotypic properties of immune cells that associate with outcome to ICI therapy, and are well aligned with current principles of immunology. Our study thus serves as an important example of the power of the ability to delineate both tumour and immune cell phenotypes, infer their functional properties and measure how their changes can associate with patient outcomes. Such techniques are placed to provide an unparalleled approach to understand how to better manage therapeutic strategies and aid diagnostic pathology.

### Supplementary Information

**Additional file 1: Fig. S1 a** Representative immunofluorescent images of TMA cores in the assay. DAPI (White), CD45 (Blue), Pan-cytokeratin (Red). **b** Representative immunofluorescent images of markers in the assay. **c** Representative immunofluorescent images of markers in the assay. **Fig. S2** **a** UMAP and TSNE plots of Phenograph clusters and assigned cell types. Heatmap of marker expression in each cluster. **b** Markers ranked by T test for enrichment within each cluster. **Fig. S3.** Representative single channel images of cell types identified.

**Additional file 2: Table S1.** Table of antibody clones, barcode, reporter, and CODEX imaging cycles.

**Additional file 3: Table S2.** T-test results for all features analysed.

**Additional file 4: Table S3.** Survival analysis by Cox proportional hazards results for all features analysed.

## Data Availability

Raw image data, segmented expression matrix and annotated Anndata are available under Zenodo DOI 10.5281/zenodo.1025857810.5281/zenodo.10258578. Analysis scripts can be found at https://github.com/clinicalomx. Feature tables and t-tests for volcano plots, PCAs, and survival were imported into R for plotting.
