# Supplementary figures and images for: Spatial insights into immunotherapy response in non-small cell lung cancer (NSCLC) by multiplexed tissue imaging
Source: J Transl Med. 2024 Mar 4;22:239. doi: 10.1186/s12967-024-05035-8 (PMC10910756; doi:10.1186/s12967-024-05035-8)

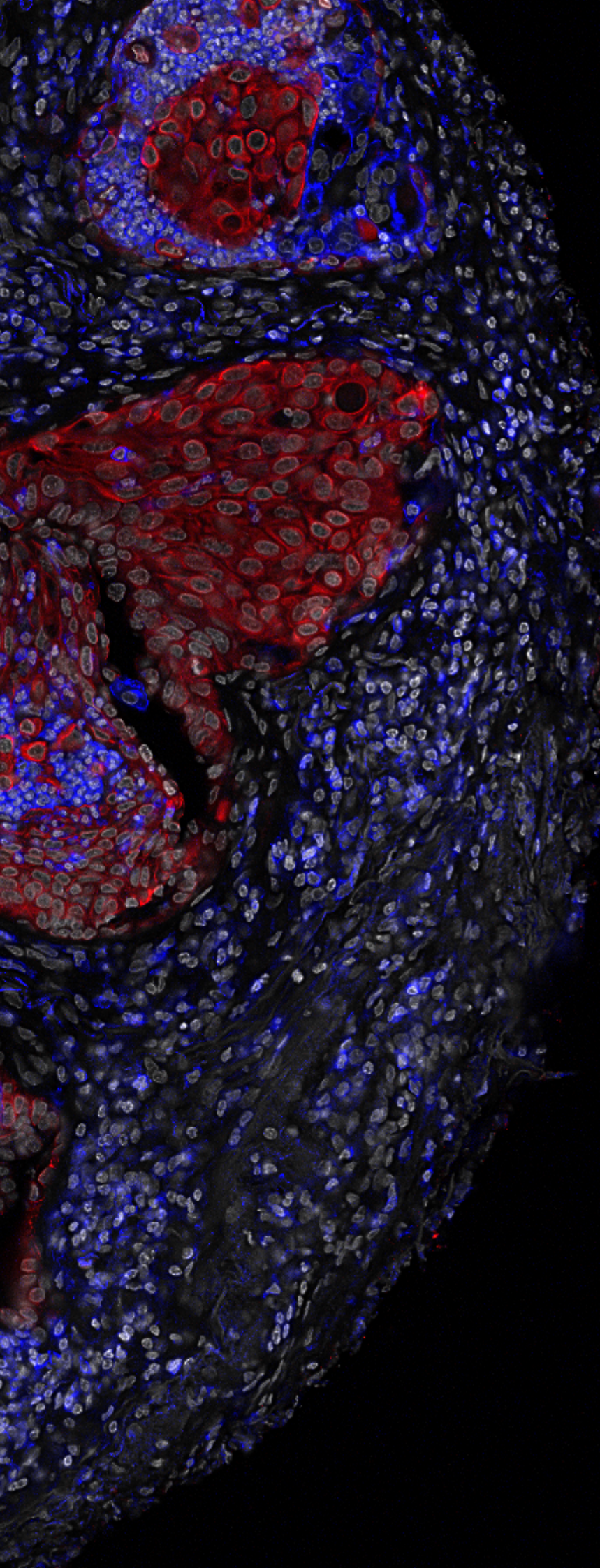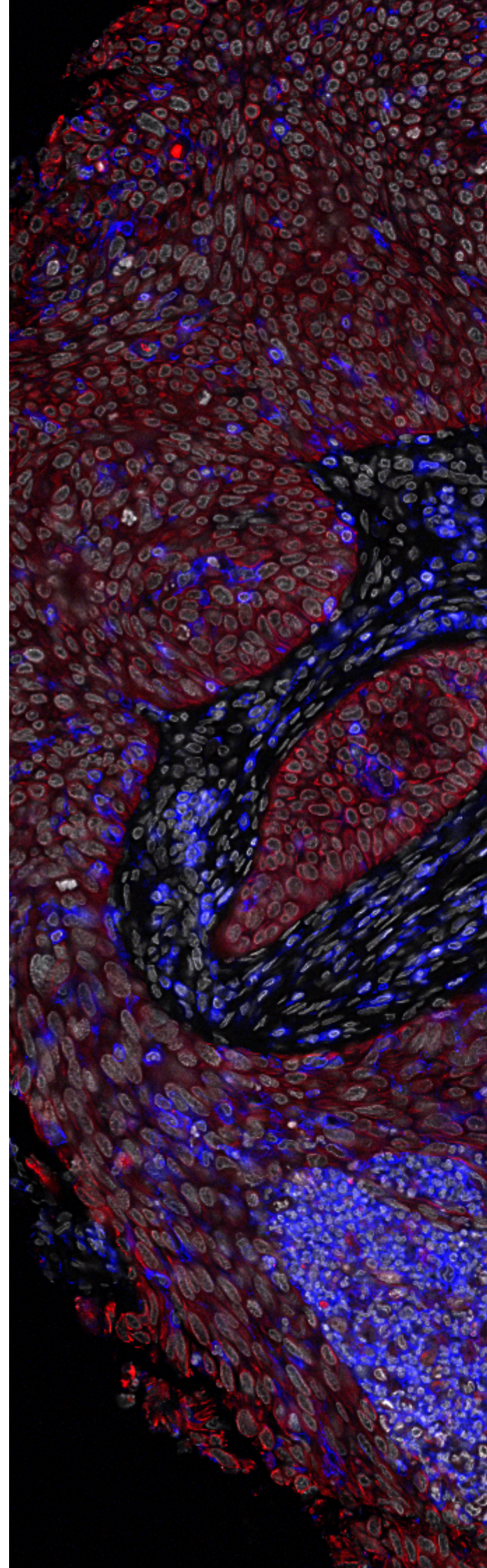

Supplement: Supplementary file 1 — Additional file 1: Fig. S1 a Representative immunofluorescent images of TMA cores in the assay. DAPI (White), CD45 (Blue), Pan-cytokeratin (Red). b Representative immunofluorescent images of markers in the assay. c Representative immunofluorescent images of markers in the assay. Fig. S2 a UMAP and TSNE plots of Phenograph clusters and assigned cell types. Heatmap of marker expression in each cluster. b Markers ranked by T test for enrichment within each cluster. Fig. S3. Representative single channel images of cell types identified. [file 12967_2024_5035_MOESM1_ESM.zip › New folder/supp1a_allcoresordered_excludedshown.pdf]

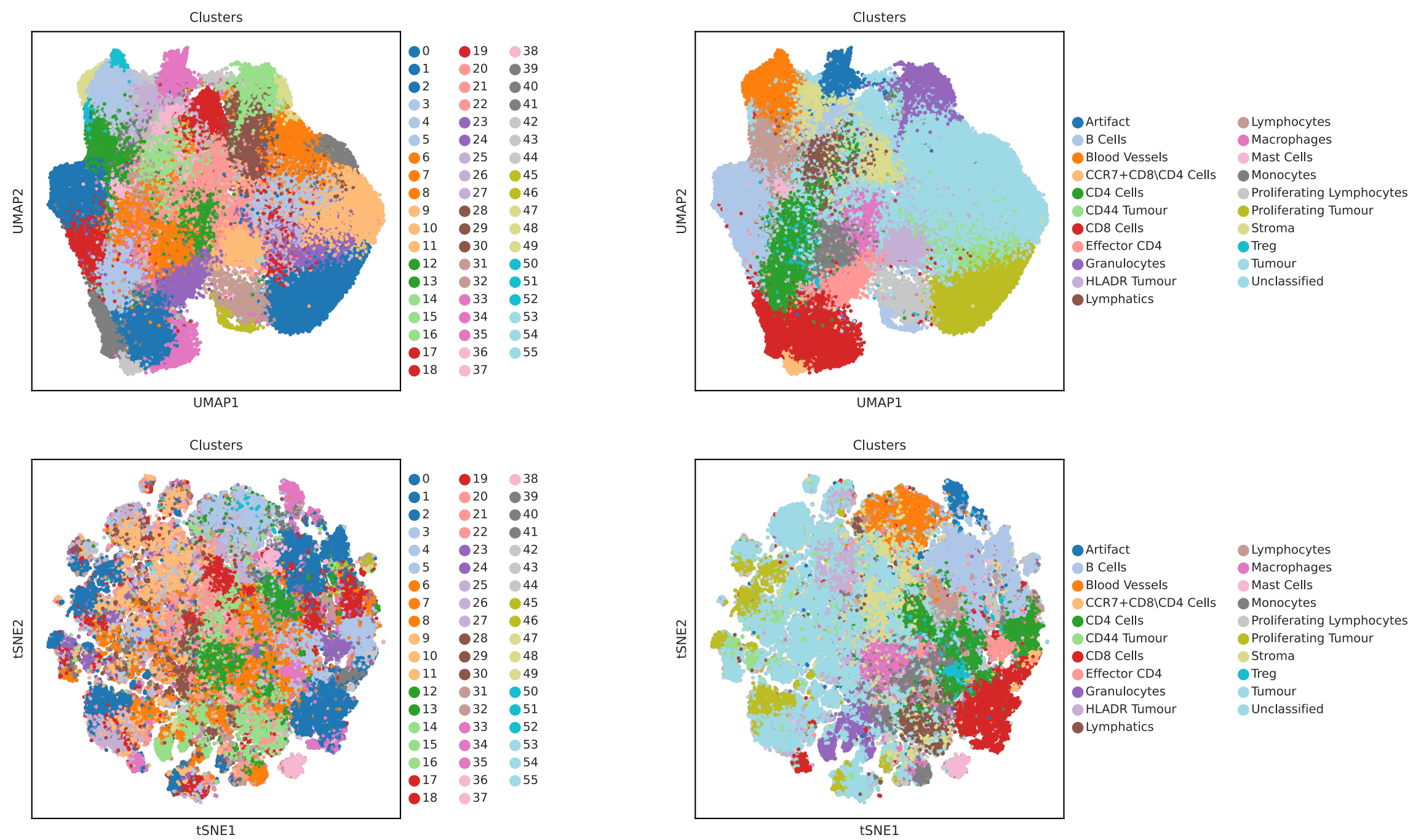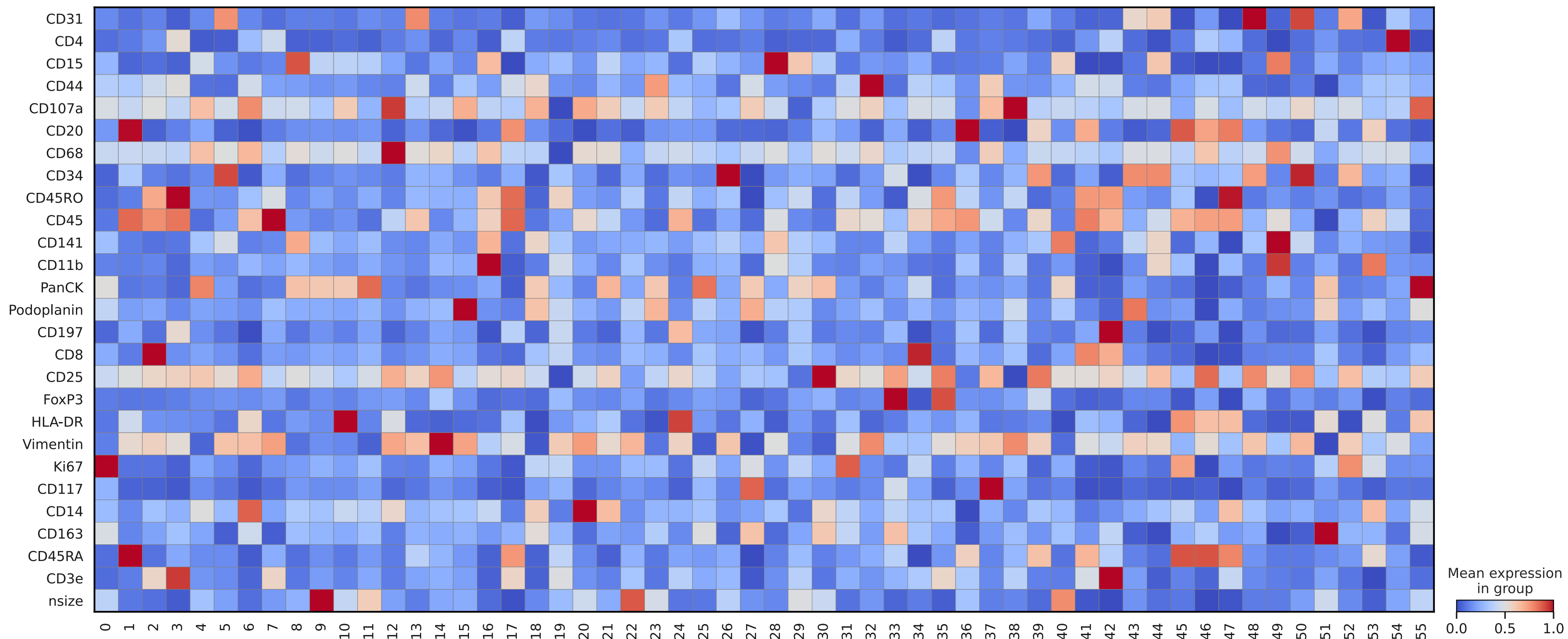

Supplement: Supplementary file 1 — Additional file 1: Fig. S1 a Representative immunofluorescent images of TMA cores in the assay. DAPI (White), CD45 (Blue), Pan-cytokeratin (Red). b Representative immunofluorescent images of markers in the assay. c Representative immunofluorescent images of markers in the assay. Fig. S2 a UMAP and TSNE plots of Phenograph clusters and assigned cell types. Heatmap of marker expression in each cluster. b Markers ranked by T test for enrichment within each cluster. Fig. S3. Representative single channel images of cell types identified. [file 12967_2024_5035_MOESM1_ESM.zip › New folder/supp2a_clustering_CT_umap.pdf]

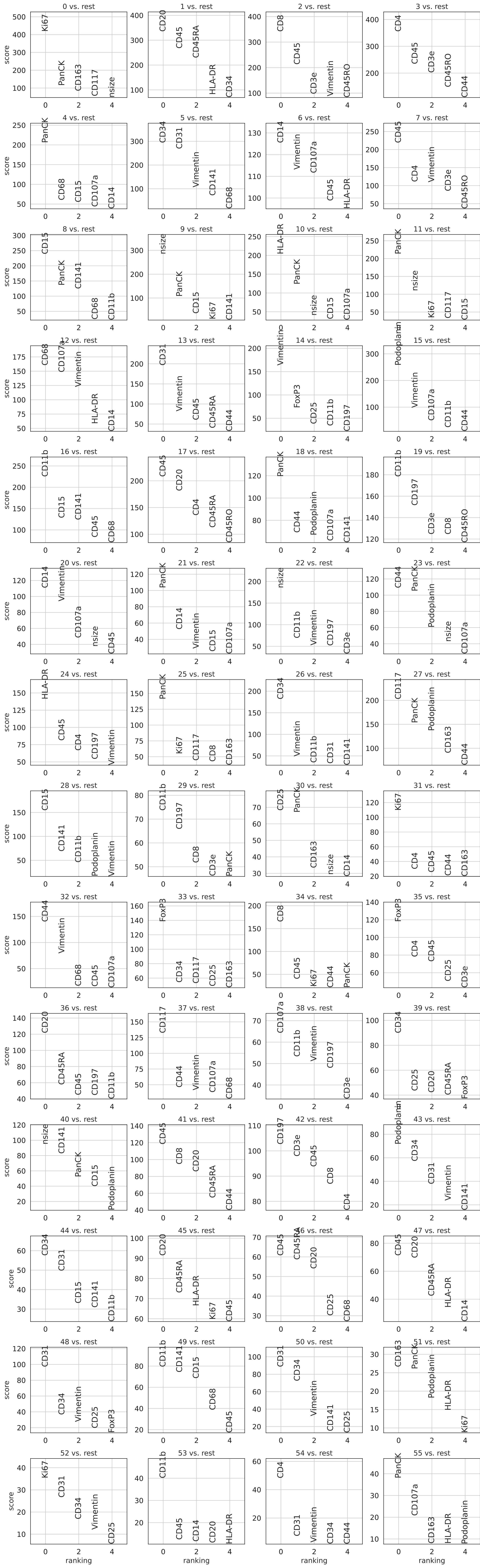

Supplement: Supplementary file 1 — Additional file 1: Fig. S1 a Representative immunofluorescent images of TMA cores in the assay. DAPI (White), CD45 (Blue), Pan-cytokeratin (Red). b Representative immunofluorescent images of markers in the assay. c Representative immunofluorescent images of markers in the assay. Fig. S2 a UMAP and TSNE plots of Phenograph clusters and assigned cell types. Heatmap of marker expression in each cluster. b Markers ranked by T test for enrichment within each cluster. Fig. S3. Representative single channel images of cell types identified. [file 12967_2024_5035_MOESM1_ESM.zip › New folder/supp2b_sc_rankgenes.pdf]
