# Supplementary material for: Spatial insights into immunotherapy response in non-small cell lung cancer (NSCLC) by multiplexed tissue imaging
Source: J Transl Med. 2024 Mar 4;22:239. doi: 10.1186/s12967-024-05035-8 (PMC10910756; doi:10.1186/s12967-024-05035-8)
Supplement: Supplementary file 1 — Additional file 1: Fig. S1 a Representative immunofluorescent images of TMA cores in the assay. DAPI (White), CD45 (Blue), Pan-cytokeratin (Red). b Representative immunofluorescent images of markers in the assay. c Representative immunofluorescent images of markers in the assay. Fig. S2 a UMAP and TSNE plots of Phenograph clusters and assigned cell types. Heatmap of marker expression in each cluster. b Markers ranked by T test for enrichment within each cluster. Fig. S3. Representative single channel images of cell types identified. [file Supp1b_markers.pdf › New folder/Supp1b_markers.pdf]

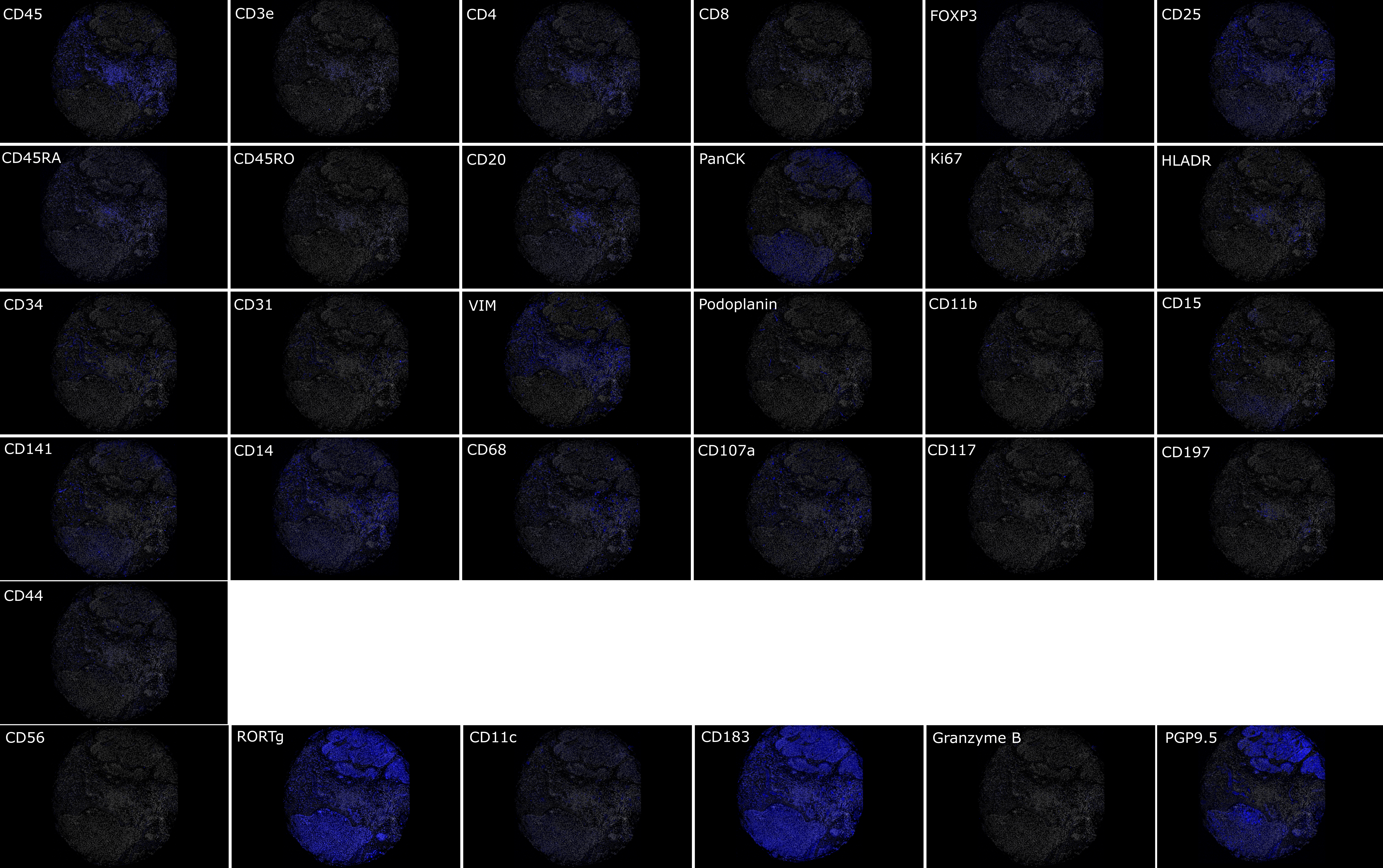
CD45
CD3e
CD4
CD8
FOXP3
CD25
CD45RA
CD45RO
CD20
PanCK
Ki67
HLADR
CD34
CD31
VIM
Podoplanin
CD11b
CD15
CD141
CD14
CD68
CD107a
CD117
CD197
CD44
CD56
RORTg
CD11c
CD183
Granzyme B
PGP9.5
