# Supplementary material for: Spatial insights into immunotherapy response in non-small cell lung cancer (NSCLC) by multiplexed tissue imaging
Source: J Transl Med. 2024 Mar 4;22:239. doi: 10.1186/s12967-024-05035-8 (PMC10910756; doi:10.1186/s12967-024-05035-8)
Supplement: Supplementary file 1 — Additional file 1: Fig. S1 a Representative immunofluorescent images of TMA cores in the assay. DAPI (White), CD45 (Blue), Pan-cytokeratin (Red). b Representative immunofluorescent images of markers in the assay. c Representative immunofluorescent images of markers in the assay. Fig. S2 a UMAP and TSNE plots of Phenograph clusters and assigned cell types. Heatmap of marker expression in each cluster. b Markers ranked by T test for enrichment within each cluster. Fig. S3. Representative single channel images of cell types identified. [file Supp3_celltypes.pdf › New folder/Supp3_celltypes.pdf]

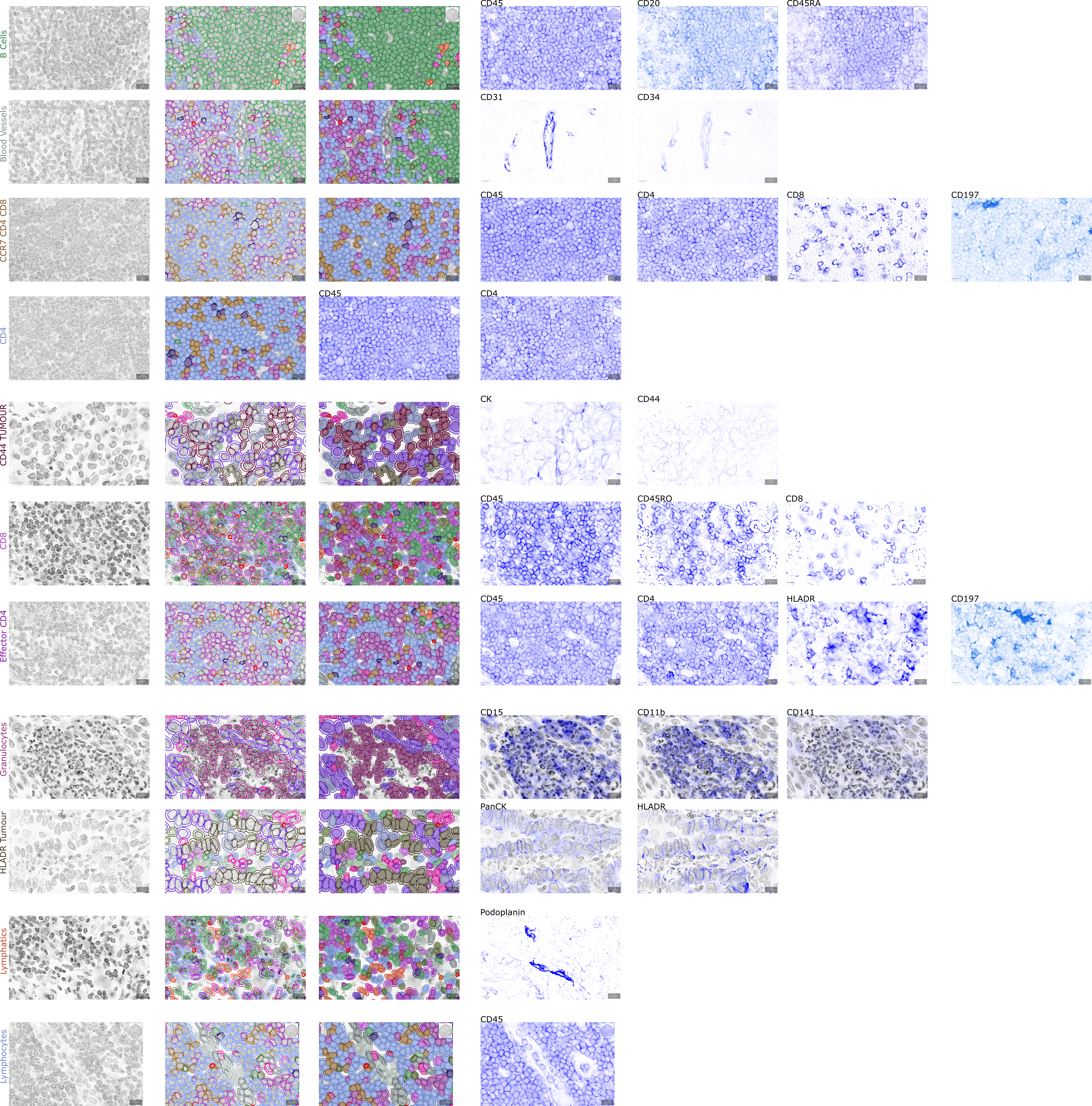

B Cells
CD45
CD20
CD45RA
Blood Vessels
CD31
CD34
CCR7 CD4 CD8
CD45
CD4
CD8
CD197
CD4
CD45
CD4
CD44 TUMOUR
CK
CD44
CD8
CD45
CD45RO
CD8
Effector CD4
CD45
CD4
HLADR
CD197
Granulocytes
CD15
CD11b
CD141
HLADR Tumour
PanCK
HLADR
Lymphatics
Podoplanin
Lymphocytes
CD45
